# Facilitation of electroporative drug uptake and cell killing by electrosensitization

**DOI:** 10.1111/j.1582-4934.2012.01658.x

**Published:** 2013-01-11

**Authors:** Olga N Pakhomova, Betsy W Gregory, Andrei G Pakhomov

**Affiliations:** Frank Reidy Research Center for Bioelectrics, Old Dominion UniversityNorfolk, VA, USA

**Keywords:** electroporation, electrochemotherapy, electric pulses, membrane permeability, ablation

## Abstract

Cell permeabilization by electric pulses (EP), or electroporation, is widely used for intracellular delivery of drugs and plasmids, as well as for tumour and tissue ablation. We found that cells pre-treated with 100-μs EP develop delayed hypersensitivity to subsequent EP applications. Sensitizing B16 and CHO cells by splitting a single train of eight 100-μs EP into two trains of four EP each (with 5-min. interval) decreased the LD_50_ 1.5–2 times. Sensitization profoundly enhanced the electroporation-assisted uptake of bleomycin, a cell-impermeable cytotoxic agent accepted for killing tumours by electrochemotherapy. EP exposures that were not lethal *per se* caused cell death in the presence of bleomycin and proportionally to its concentration. Sensitizing cells by a split-dose EP exposure increased bleomycin-mediated lethality to the same extent as a 10-fold increase in bleomycin concentration when using a single EP dose. Likewise, sensitization by a split-dose EP exposure (without changing the overall dose, pulse number, or amplitude) enhanced the electroporative uptake of propidium up to fivefold. Enhancement of the electroporative uptake appears a key mechanism of electrosensitization and may benefit electrochemotherapy and numerous applications that employ EP for cell permeabilization.

## Introduction

Electropermeabilization by intense electric pulses (EP), also commonly known as electroporation is a well-established physical method of disrupting cell membrane, to kill cells or to facilitate the uptake of membrane-impermeable substances without cell killing [1–4]. This method is central for many existing and emergent medical applications, including tissue and tumour ablation by irreversible electroporation (IRE) [2, 4–7], electrochemotherapy (ECT) [4, 8–11], gene electrotransfer [12–17], and decellularization of transplants [18].

Recently, we found that cells subjected to electroporation develop delayed sensitization to EP, and that the cytotoxic effect can be markedly increased by splitting a single EP treatment into two fractions [19]. The cell death increases because the first fraction not only incurs cell damage but also induces delayed electrosensitization, thereby profoundly enhancing the effect of the second fraction and of the treatment as a whole. By engaging sensitization, the lethality in EP-treated cells could be increased from 0% to 90%, or the exposure dose could be reduced more than twofold without reducing the effect. Thus far, sensitization has been reported *in vitro* for U937, Jurkat, and CHO cells using EP of 60-ns to 9-μs duration, at 1.8–13.3 kV/cm. However, the mechanism responsible for the phenomenon of sensitization has not been understood.

Contemplated mechanisms include cell swelling and plasma membrane spreading; ATP leakage and exhaustion of membrane repair resources; electrochemical generation of ROS and membrane damage by ROS; entry of Ca^2+^ and triggering the respective intracellular cascades; and several others. The central question is whether the membrane of sensitized cells is permeabilized more efficiently, allowing greater uptake of substances (drugs, plasmids, siRNA) without increasing the EP dose or intensity. If this is the case, engaging sensitization by split-dose EP treatment protocols could benefit numerous technologies that rely on electroporation for intracellular delivery and medical applications such as ECT.

Electrochemotherapy exploits EP treatments which are sufficient for cell membrane permeabilization, but cause little cell death *per se*. ECT relies on the electroporative uptake of a cell-impermeable or poorly permeable cytotoxic drug, such as bleomycin or cisplatin. Local uptake of the drug in the area of EP application ensures elimination of dividing cells [8–11, 20–22]. More than 40 different types of tumours responded to ECT, including those incurable with chemotherapy and not suitable for excision surgery. In clinical applications, ECT caused complete regression of 75–80% of treated nodules. The established and clinically approved EP delivery protocol for ECT consists of eight pulses of 100-μs duration, which are delivered at either 1 or 1000 Hz [8, 11].

Although EP treatments are minimally invasive and efficient, they may cause severe pain, damage of healthy tissues surrounding the ablation area, involuntary muscle contractions and heart fibrillation [23–25]. Use of a split-dose protocol to sensitize cells could potentially help to achieve the same electroporative drug uptake at lower EP amplitude, thereby profoundly reducing the side effects while maintaining the treatment efficiency. Below, we show that enhanced membrane permeabilization accompanies electrosensitization and likely constitutes its principle mechanism. We show that a split-dose delivery of 100-μs EP efficiently caused electrosensitization in melanoma and epithelial cell models, and that the sensitized cells responded to EP by profoundly higher electroporative uptake of bleomycin. Likewise, electrosensitized cells displayed higher uptake of propidium, a fluorescent marker that does not penetrate intact cell plasma membrane.

## Materials and methods

### Cell lines

CHO-K1 (Chinese hamster ovary) and B16.F10 (mouse melanoma) cells were obtained from ATCC (Manassas, VA, USA) and grown in humidified 5% CO_2_ in air in standard culture dishes. CHO cells were propagated in AMEM medium supplemented with 100 IU/ml penicillin and 0.1 μg/ml streptomycin. B16 cells were propagated in McCoy's 5A medium supplemented with 0.01% gentamicin. Both growth media contained 10% foetal bovine serum. The media and its components were purchased from Mediatech Cellgro (Herdon, VA, USA) except for serum (Atlanta Biologicals, Norcross, GA, USA).

### EP exposure, dosimetry, and thermometry

The pulse duration, number of pulses and inter-pulse intervals were set using an S88K stimulator (Grass Instruments Co., Quincy, MA, USA). These pulses gated a custom-made high-voltage, low-output impedance electroporator device, which replicated stimulator pulses and delivered them to an electroporation cuvette with a cell sample. In this study, we used nearly rectangular 100-μs pulses with 10-ms interpulse interval, and delivered them either as a single train of eight pulses (single dose), or as two trains of four pulses each (split dose). These exposure parameters were chosen to match the standard ECT protocol [8, 9, 11] as closely as was technically possible, to facilitate possible translation of our results into clinical practice. For fractionated exposures, the 50/50 split (4+4 pulses) and the 5 min. interval between the fractions were suggested in our earlier study [19]. The pulse shape and amplitude were monitored with a TDS3052B oscilloscope (Tektronix, Wilsonville, OR, USA). The E-field values were obtained by dividing the mean pulse voltage (from 50 to 300 V) by the width of the gap in the electroporation cuvette (1 mm). The absorbed dose was calculated as the energy delivered to the sample normalized to the mass of the sample [26]; the maximum tested dose was 60 J/g.

All EP exposures were performed at a room temperature of 22–24°C. Heating of cell samples by EP was measured with a fibre optic ReFlex-4 thermometer (Nortech Fibronic, Quebec City, Quebec, Canada). Because of the efficient heat dissipation from the cuvette, the temperature of exposed samples did not exceed 37°C even at the maximum EP dose.

### EP Cytotoxicity

Cells were harvested during the exponential growth phase, pelleted by mild centrifugation, and resuspended at 3 × 10^6^ cells/ml in fresh growth medium. The cell suspension was loaded into a pair of standard electroporation cuvettes (BioSmith Biotech, San Diego, CA, USA) and exposed to EP within several minutes. Both cuvettes received the same EP treatment, but it was split into two fractions for one of the cuvettes. Although the single-dose exposure took less than a second and the split-dose one took 5 min., both samples remained in the cuvettes until both exposures were completed. Immediately afterwards, the samples were aliquoted into microcentrifuge tubes and diluted with the fresh growth medium to 0.15 × 10^6^ cells/ml. The experiment continued the same way with a next pair of cuvettes and testing a different E-field, and so forth. In each experiment, different E-field levels were tested in a random sequence; the first and the last pair in each experiment were accompanied by a third cuvette that was subjected to a sham exposure (control).

In each experiment, we tested five different E-field values (from 0 to 3 kV/cm). Upon the completion of all exposures, cells were aseptically aliquoted from the tubes into a 96-well plate, in triplicates at 15 × 10^3^ cells/well and incubated at 37°C with 5% CO_2_ in air. Cell survival was measured using the 3-(4,5-Dimethylthiazol-2-yl)-2,5-diphenyltetrazolium bromide (MTT) assay (BioAssay Systems, Hayward, CA, USA). At 20 hrs after the EP treatment, 10 μl of MTT reagent was added to each well, and incubation continued for additional 3 hrs. Next, the medium from the wells was aspirated and replaced with 100 μl DMSO, and the plate was placed on an orbital shaker for 10 min. to dissolve blue formazan crystals. Absorbance at 570 nm was read using Synergy 2 microplate reader (BioTEK, Winooski, VT, USA), and the readings in EP-exposed samples were normalized to the sham-exposed control.

### Electroporative uptake of bleomycin

In a separate series of experiments, cells were subjected to single- and split-dose EP exposures in the presence of different concentrations of bleomycin. Lyophilized bleomycin (Sigma-Aldrich, St. Louis, MO, USA) was dissolved in a phosphate-buffered saline (PBS) at 5 mg/ml (3.5 mM) and stored at −20°C. Bleomycin was added to the cell suspension immediately prior to exposures at the concentration of 0.058, 0.54, 5.9, or 65 μM. The suspension was dispensed in three electroporation cuvettes: one for a single-dose exposure, the second one for a split-dose exposure, and the third cuvette served as a parallel control (sham exposure). The single-dose exposure was always performed before the split-dose exposure, which deliberately biased the results towards possibly reduced effect of sensitization (discussed in more detail in the Results section). The E-field amplitude was fixed at a low value of 1.5 kV/cm, which caused little or no cell death in the absence of the drug. Immediately after the exposures, the samples were aliquoted into microcentrifuge tubes and incubated on a bench for additional 10 min., and then diluted 20-fold with the fresh growth medium. The experiment continued the same way with a next triplet and using the same E-field, but testing a different drug concentration. In each experiment, different bleomycin concentrations were tested in a random sequence, and were also alternated with the controls where no drug was added. The cell survival was measured next day by the MTT assay the same way as described above.

### Electroporative uptake of propidium

On the day of the experiment, harvested cells were resuspended in a fresh growth medium and dispensed into 35-mm Petri dishes (0.6 × 10^6^ cells in 2 ml). The dishes were moved to the incubator for a minimum of 1 hr. Immediately prior to the experiment, cells from one Petri dish were collected, pelleted, and resuspended in 0.6 ml of a physiological solution containing (in mM): 140 NaCl, 5.4 KCl, 2 CaCl_2_, 1.5 MgCl_2_, 10 glucose and 10 HEPES (pH 7.3, 300 mOsm/kg). Propidium (Pr) iodide was added to the suspension at 20 μg/ml, and 140-μl samples were dispensed into three electroporation cuvettes. Exposures were performed the same way as described in the previous section (single dose, split dose and sham exposure). Immediately after the exposures, the samples were aliquoted in individual wells of a 96-well plate. Pr fluorescence (exc./em.: 530/590 nm) was read every 5 min. for the next 30 min. with the microplate reader. Pr uptake data were expressed as percentage of the parallel positive control (a sample of the same cell suspension lysed with 0.05 mg/ml digitonin). All chemicals were purchased from Sigma-Aldrich.

## Results and discussion

### Split-dose protocol facilitates the cytotoxic effect of 100-μs pulses

**Fig. 1:**
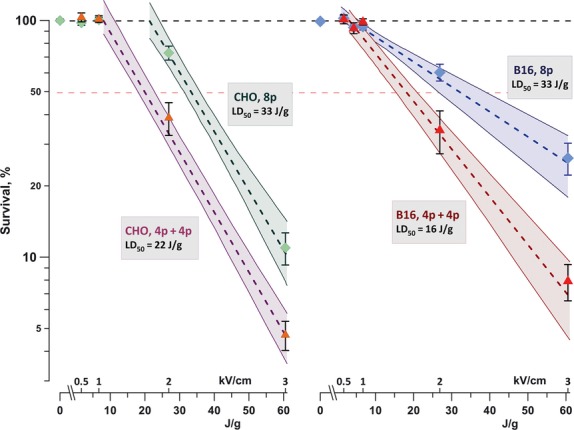
Enhancement of the cytotoxic effect of 100-μs electric pulses (EP) by exposure fractionation. CHO cells (left panel) and B16 cells (right panel) were exposed to eight pulses (100 Hz) delivered either as a single dose (8p) or a split dose (4p+4p) with 5-min. interval between two trains. The graphs show cell survival (mean ± SE for three to six independent experiments) *versus* the dose for different EP treatments. Dashed lines are the best fit data approximations using exponential function; shaded areas denote 95% confidence intervals. Cell survival was measured by MTT assay at 24 hrs post exposure. Legends show lethal dose values for elimination of 50% of cells (LD_50_) by the respective exposure protocols.

In both CHO and B16 cells, exposure to eight 100-μs pulses at 2 or 3 kV/cm significantly decreased the 24-hr cell survival (Fig. 1). Similarly to what was reported previously for shorter EP [19, 27–29], cell survival curves started with a ‘shoulder’ (no cell death), followed by an exponential decrease proportionally to the absorbed dose. The efficiency of different EP treatments could be conveniently compared using the width of the shoulder and the dose that killed 50% of cells (LD_50_).

In CHO cells, using a split-dose protocol decreased LD_50_ 1.5 times (from 33 to 22 J/g) and reduced the shoulder from 22 to 8 J/g. In B16 cells, the shoulder was not affected, but the LD_50_ was reduced twofold. Overall, the enhancement of the cytotoxic effect by sensitization was similar to what was reported previously in other cell lines and using nanosecond-range EP [19].

### Sensitization assists electroporative uptake of bleomycin

**Fig. 2:**
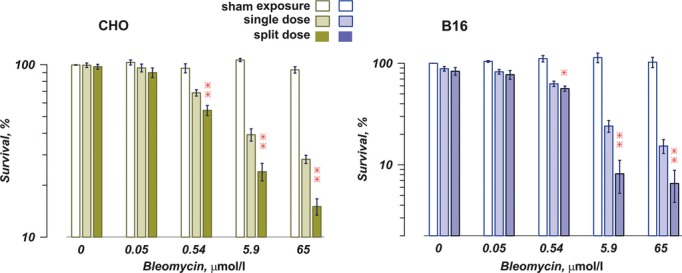
Sensitization of cells by a split-dose electric pulses (EP) delivery facilitates electroporative uptake of bleomycin. CHO cells (left panel) and B16 cells (right panel) were exposed to eight, 100-μs pulses (100 Hz, 1.5 kV/cm), either as a single dose or as a split dose (two trains of four pulses each with a 5-min. interval). The graphs show cell survival in 24 hrs after the treatment (mean ± SE, *n* = 3–5) Bleomycin was added to the medium at concentrations shown in the graph. EP caused little cell death in the absence of bleomycin, but facilitated the drug uptake and cell death proportionally to bleomycin concentrations. The split-dose exposure triggered significantly higher bleomycin uptake than the single dose (*P < 0.05, **P < 0.01, 2-tailed paired *t*-test). See text for more details.

In the absence of bleomycin, exposure to eight pulses at 1.5 kV/cm caused no detectable cell killing (CHO cells) or decreased cell survival by just 5–10% (B16). When cells were similarly electroporated in the presence of bleomycin, their survival was decreased proportionally to the concentration of the drug in the medium (Fig. 2). Notably, the survival of control cells that were not exposed to EP was not reduced even by the highest concentration of bleomycin, so the reduction in cell survival could be attributed completely (CHO) or mostly (B16) to the electroporative uptake of this cytotoxic drug.

The split-dose EP exposure triggered profoundly higher bleomycin uptake than the single dose. For example, with 5.9 μM of bleomycin in the medium, sensitizing cells by the split-dose EP protocol increased the bleomycin-mediated lethality to the same or greater extent than was achieved by a single-dose exposure with 65 μM of bleomycin. In other words, sensitization of cells resulted in more than 10-fold increase in the electroporative uptake of bleomycin without changing the E-field or the number of pulses.

The split-dose exposure was more efficient even despite the fact that during the first 5 min. of incubation with bleomycin the cells were porated by only four EP and obviously were taking up lesser drugs than the cells exposed to all eight pulses as a single dose. For a fair comparison, the split-dose exposed cells should have been incubated longer, but the exact additional time was impossible to define. To be on the conservative side, we used the protocol biased against the effect of sensitization (single-dose exposure first, split-dose second), hoping that sensitization will be strong enough to overcome the bias. Indeed, the data show that after receiving the second EP fraction, the split-dose exposed cells accumulated more than enough bleomycin to compensate for its lower uptake during the first 5 min.

### Sensitization facilitates early uptake of propidium

Fluorescence detection of Pr uptake is a common method to distinguish live and dead cells; however, disruption of cell membrane by electroporation may trigger transient uptake of the dye, which is not necessarily indicative of the lethal cell damage. In electroporated cells, the early uptake of Pr reflects the degree of membrane permeabilization. If the cell fails to repair the membrane, it swells until membrane rupture and death, which is reflected by delayed (minutes) acceleration in Pr uptake [19, 30].

**Fig. 3:**
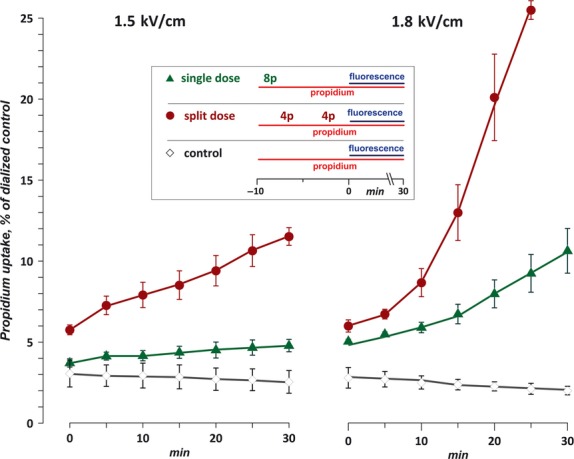
Enhancement of propidium (Pr) uptake by CHO cells by electrosensitization. The cells were exposed to eight, 100-μs pulses (100 Hz) at either 1.5 kV/cm (left panel) or 1.8 kV/cm (right). The treatment was performed either as a single dose or as a split dose (two trains of four pulses each with a 5-min. interval). Pr fluorescence (mean ± SE, *n* = 3) was normalized to the value in a sample dialyzed with digitonin. The inset shows the timeline of procedures in each of the plotted groups (‘8p’ and ‘4p’ show the approximate time of exposure to eight electric pulses (EP) and four EP, respectively; horizontal lines denote the period of incubation with propidium iodide and the time interval when fluorescence was measured). The split dose protocol caused significantly higher Pr uptake than a single dose for all time-points except 0 min. at 1.8 kV/cm (*P* < 0.05–0.0001, 2-tailed paired *t*-test). See text for more detail.

Figure 3 shows that Pr uptake early after exposure was significantly higher in cells exposed using the split-dose protocol. The difference from the single-dose exposure was particularly well seen with the low E-field exposure (1.5 kV/cm), which caused little or no cell death. These experiments were also designed conservatively (same as described for bleomycin above), so the data in Fig. 3 actually underestimate the impact of sensitization. Indeed, prior to the onset of measurements, the single-dose exposed cells were taking up Pr for about 8 min. after eight EP; the split-dose exposed cells were taking up Pr for only 2 min. after eight EP, plus for 5 min. after the first four EP (Fig. 3, inset). If the first train of four EP did not cause sensitization, the Pr uptake in the split-dose exposed group would likely be much lower than that in the single-dose group.

### Summary: Enhanced electroporation as a key feature of electrosensitization

Both in Pr and bleomycin experiments, the first train of four EP obviously caused weaker membrane permeabilization and less substance uptake than a similar train of eight EP. However, the second train of four EP caused high substance uptake, which quickly overwhelmed the previous 5-min. lag. These data suggest that it is more efficient plasma membrane permeabilization that distinguishes sensitized cells, and that this change in membrane properties is the hallmark of sensitization. The sensitized membrane may respond to EP by forming more pores, larger pores, slower pore resealing, or any combination of the above. Future search for mechanisms responsible for electrosensitization should be focused on how ‘priming’ of cells by EP modifies the plasma membrane to make it hypersensitive to electroporation.

Even before the exact mechanisms of sensitization are established, taking this phenomenon into account may benefit multiple EP applications that rely on electroporation for intracellular delivery (transfection by electroporation, gene electrotransfer, loading cells with drugs and siRNA, etc.) To pick up just one, in this article we focused on the EP protocols accepted for ECT and found that sensitization is promising for increasing the ECT efficiency while reducing its side effects. This observation now needs to be extended to 3D cell models and *in vivo*, along with further analysis of its underlying mechanisms.
